# Association between Dietary Patterns of Meat and Fish Consumption with Bone Mineral Density or Fracture Risk: A Systematic Literature

**DOI:** 10.3390/nu9091029

**Published:** 2017-09-18

**Authors:** Simone Perna, Ilaria Avanzato, Mara Nichetti, Giuseppe D’Antona, Massimo Negro, Mariangela Rondanelli

**Affiliations:** 1 Department of Public Health, Experimental and Forensic Medicine, School of Medicine, Endocrinology and Nutrition Unit, University of Pavia, Azienda di Servizi alla Persona di Pavia, Pavia 27100, Italy; ilak_a@yahoo.it (I.A.); dietista.mara.nichetti@gmail.com (Mar.N.); mariangela.rondanelli@unipv.it (M.R.); 2 CRIAMS-Sport Medicine Centre, University of Pavia, Voghera 27058, Italy; gdantona@unipv.it; 3 Department of Public Health, Experimental and Forensic Medicine, University of Pavia, Pavia 27100, Italy

**Keywords:** meat, fish, osteoporosis, fractures, bone, Asian, Mediterranean, diet, animal proteins

## Abstract

This systematic review aimed to investigate the association of fish and sea fish dietary patterns (FishDiet) and meat or processed meat dietary patterns (MeatDiet) with bone mineral density (BMD) and/or risk of fractures (RF). This review includes 37 studies with a total of 432,924 subjects. The results suggest that MeatDiet and FishDiet did not affect BMD or RF in 48.2% of the subjects with MeatDiet and in 86.5% of the subjects with FishDiet. Positive effects on bone were found in 3% of subjects with MeatDiet and in 12% with FishDiet. Negative effects on bone were observed in 2.7% of FishDiet and in 47.9% of MeatDiet. Major negative effects of MeatDiet were found in subjects located in the Netherlands, Greece, Germany, Italy, Norway, UK and Spain who do not sustain a Mediterranean diet (92.7%); in Korea (27.1%); in Brazil and Mexico (96.4%); and in Australia (62.5%). This study suggests that protein intake from fish or meat is not harmful to bone. Negative effects on bone linked to FishDiet are almost null. Negative effects on bone were associated to MeatDiet in the setting of a Western Diet but not in Mediterranean or Asian Diets.

## 1. Introduction

Primary osteoporosis prevention requires healthy behaviour, such as regular physical exercise, and adequate dietary intake of calcium, vitamin D and proteins [1]. In particular, proteins (derived from meat, fish, milk and eggs) are essential because they are incorporated into the organic matrix of bone as part of the collagen structure upon which mineralization occurs, and because dietary proteins influence the secretion and action of the osteotropic hormone insulin-like growth factor I (IGF-I), which is important for bone formation [2]. Minerals (in particular calcium and phosphorus because they compose roughly 80% to 90% of the mineral content of bone hydroxyapatite) and vitamins (e.g., vitamins D and K) are also crucial for carrying out metabolic processes and reactions in bone [3]. 

Other benefits for bone metabolism are derived from bioactive components found predominantly in vegetables, but also in some herbs and fruits: phytochemicals, antioxidants, and other bioactive compounds influence bone metabolism through a variety of mechanisms, but mainly through reducing oxidative stress and inflammation [4]. In particular, cellular studies on dried plum, citrus, berry fruits and bioactive compounds including lycopene, phenolics, flavonoids, resveratrol, phloridzin, isoflavones and pectin derived from tomato, grapes, apples, and citrus fruits seem to be promising. Furthermore, animal studies strongly suggest that commonly consumed antioxidant-rich fruits have a pronounced effect on trabecular bone volume, number, and thickness, and lower trabecular separation through enhancing bone formation and suppressing bone resorption [5].

Several studies have concluded that the incidence of osteoporosis and osteoporosis-related fractures vary across the European Union. Conspicuous differences are encountered in the incidence of osteoporosis, the lowest incidence being reported in the Mediterranean area [6]. The beneficial effect is primarily attributed to a specific pattern of eating habits that includes high consumption of vegetables, legumes, fruits, and grains; moderate to high intake of fish; low intake of saturated lipids; high intake of unsaturated lipids, particularly olive oil; low to moderate intake of dairy products; low intake of meat; and modest intake of alcohol mostly in the form of wine [7,8].

The most consistently followed approach to examine the potential relationship between dietary factors and skeletal health has been based on studies of particular nutrients, such as calcium and vitamin D. Although previous studies have mainly focused on the roles of calcium, vitamin D, protein, and dairy and soy products, increasing evidence suggests a positive association between fruit and vegetable components and bone health [9,10,11]. These components include potassium; manganese; vitamin B complex; vitamins C, E, and K; and phytochemicals (e.g., carotenoids, and genistein aglycone) [11,12]. Specifically, a recent study showed that genistein aglycone administration continued to decrease levels of bone resorption markers (pyrrolidonyl aminopeptidase (PYR), telopeptide of type I collagen and receptor activator of nuclear factor κ B (RANKL)) and increased new bone formation markers (insulin growth factor (IGF-I) and osteoprotegerin (OPG)), extending this effect to three years, and supporting greater bone formation [13].

The aim of this systematic review is to investigate the effect of dietary consumption of fish and meat (or their derivatives) on bone mineral density (BMD) in studies which evaluated the association between dietary pattern and bone mineral density and/or risk of fractures as the primary outcome.

## 2. Methods

This systematic review was performed according to the following steps suggested by Egger et al. [14]:

(i) formulation of the revision question on the basis of considerations made in the abstract; and
(ii) identification of relevant studies.

The search involved all cross sectional or longitudinal cohort studies published from 1 January 1958 to 31 March 2017. English written articles were identified by searching the Medline database [15], Scopus [16], Web of Science [17] and Google Scholar [18]. The analysis was carried out in the form of a systematic review of the reports.

### 2.1. Inclusion and Exclusion Criteria

Two reviewers (SP and MR) independently reviewed each report. For each of the relevant abstracts, full publications were retrieved for evaluation based on criteria established a priori. Original cross-sectional surveys or longitudinal cohort studies investigating the effects of meat or fish as dietary patterns in relation to BMD and risk of fractures (FR) were evaluated.

As suggested by Recommendations of the World Health Organization Task-Force for Osteoporosis, the subjects with BMD values more than 2.5 standard deviations below the young normal mean should be offered appropriate treatment but intervention can also be directed at menopausal women with BMD values between −1 and −2.5 standard deviation (SD) because of their increased future fracture risk, as well as to those with other risk factors [19].

Currently, the accepted “gold standard” method for bone mineral density (BMD) measurement and osteoporosis diagnosis is dual-energy X-ray absorptiometry. In addition the quantitative ultrasound” (QUS) approaches, which are radiation-free, cheaper and portable, but they cannot be applied on the reference anatomical sites (lumbar spine and proximal femur) [20]. Changes in BMD T-score and FR were the primary outcome. No secondary outcomes were considered.

The eligible studies were required to report baseline and follow-up values of BMD, i.e., bone mass increase/decrease during the quartile (by years) during the survey, the correlation coefficient between dietary pattern and BMD. Trials were not included.

**Figure 1:**
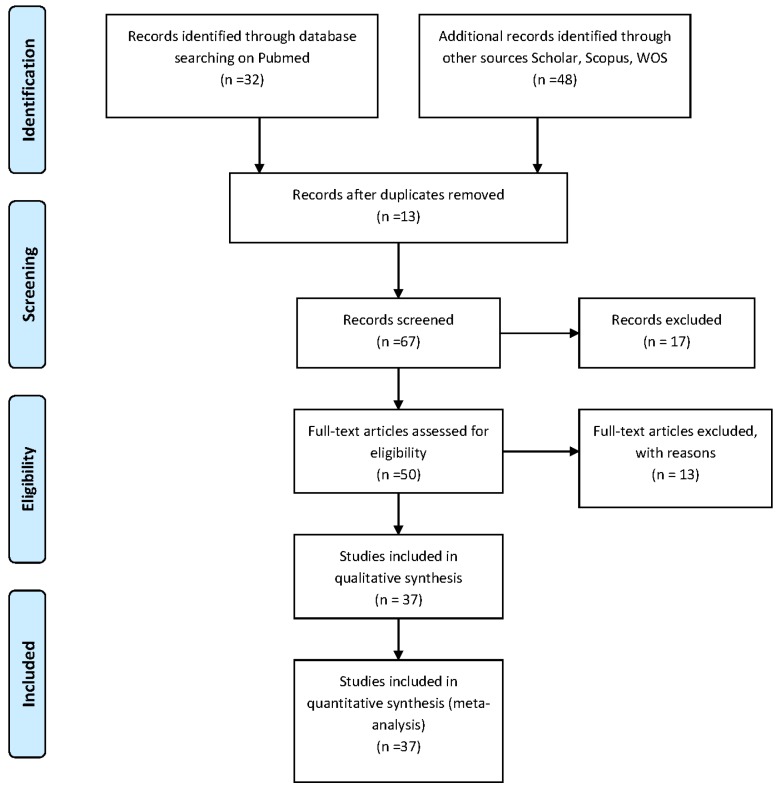
Flow diagram of the review process. WOS, web of science.

Figure 1 reports the flow diagram of the study.

### 2.2. Data Collection

The following data were extrapolated from all the revised studies:

(i) Author and year of publication;
(ii) Number of participants for each study;
(iii) Mean age of the subjects;
(iv) Country;
(v) Dietary patterns (for fish and meat consumption);
(vi) Duration of intervention (in weeks or years);
(vii) Association with BMD and Fractures Risk outcome;
(viii) Results;
(ix) Conclusions; and
(x) Effect on BMD; and Fracture Risk.

The data obtained are summarized in Table 1.

## 3. Results

Regarding the association between meat and fish dietary patterns and BMD or risk of fractures, this search was based on the keywords (“Meat” OR “Fish” OR “dietary patterns”) AND (“BMD” OR “bone mineral density” OR “osteoporosis” OR “risk of fractures”) and it retrieved 80 articles. After screening, 67 papers were selected for full-text revision. After applying the inclusion and exclusion criteria, 30 studies were excluded and 37 studies were selected for the present systematic review.

The 37 studies included a total of 432,924 subjects. Concerning study design, among the 37 studies, one was a retrospective cohort study [21], 15 were cross-sectional study [22,26,27,29,31,32,34,40,42,44,45,47,53,54,55], seven were longitudinal study [23,24,28,39,48,49,50], one was a co-twin control study [25], four were prospective cohort studies [30,33,38,51], three were population based cohort studies [35,36,43], one was “The North West Adelaide Health study” (NWAHS) [46], one was 1:1 matched case-control study [37], one was an observational study (WHI-OS) [41], one was the “Japanese Multi-Centered Environmental Toxicant Study (JMETS)” [52], one was “The Nurses’ Health Study and the Health Professionals Follow-up Study” [56], and one was “the European Prospective Investigation into Cancer and Nutrition Study” [7].

The average duration of the studies reviewed was about four years (from a minimum of one month to a maximum of 11 years) and the age range of the subjects analysed was 3–80 years. Seventeen studies considered a cohort of men and women (341,914 subjects) [7,21,23,27,35,36,37,42,43,46,48,49,50,51,53,55,56]; 16 studies considered a cohort of only women (27,255 subjects) [22,25,26,28,29,30,31,32,33,34,40,41,44,47,52,54]; one study considered a cohort of only men (1319 subjects) [45]; and 10 studies did not specify the gender of the subjects involved [24,38,39,43,46,48,49,50,51,53]. Six studies [24,35,36,38,39,53] involved children and adolescents (total of 58,891 subjects). Twenty-six studies [7,21,22,23,25,26,27,28,30,31,32,34,37,40,41,42,43,44,46,47,48,51,52,54,55,56] considered adults and elderly subjects (inclusion criteria with age ≥50 years); six studies [33,42,47,49,50,52] considered only adults (aged 21–50 years); eight studies [34,35,36,38,39,42,45,53] considered adolescents (aged 13–20 years); and one study [24] considered children (aged 4–8 years).

As regards the primary purpose of the investigations, 23 studies [22,23,24,25,26,27,29,31,32,33,34,35,36,39,42,43,44,47,48,49,50,52,54] compared the increase/decrease of BMD or increase/decrease of risk of fractures (in terms of Odds or Risk Ratio) according to FishDiet and MeatDiet. Heterogeneous associations between intake of FishDiet and MeatDiet, and risk of fractures were observed across 16 analytical epidemiologic studies [7,21,26,28,30,33,37,38,40,41,45,46,51,53,55,56] with an increase or decrease in the risk of fractures. Thirty-four studies [7,21,23,24,25,26,27,28,29,30,32,33,34,35,36,37,38,39,40,41,42,43,44,45,46,47,48,49,50,51,52,53,55,56] included MeatDiet as a dietary pattern BMD/risk of fractures for a total of 422,329. In this population, 15,712 subjects (3.7%) showed a positive effect in terms of fracture risk reduction or BMD increase. In total, 204,012 subjects (48.2) showed negative effects on bone, while no significant effects were observed in 202,605 subjects (47.9%). Thirty-two studies [7,22,23,24,25,26,27,28,30,32,33,34,35,36,37,38,39,41,42,43,44,45,46,47,48,49,50,51,52,53,54] investigated the relationship between FishDiet as a dietary pattern and BMD/risk of fractures for a total of 309,917 subjects. There was a positive effect in terms of fracture risk reduction or increase of BMD for 39,857 subjects (12%). Negative effects were found in 8570 subjects (2.7%), while in 261,490 subjects (85.3%) no significant effects were found.

**Figure 2:**
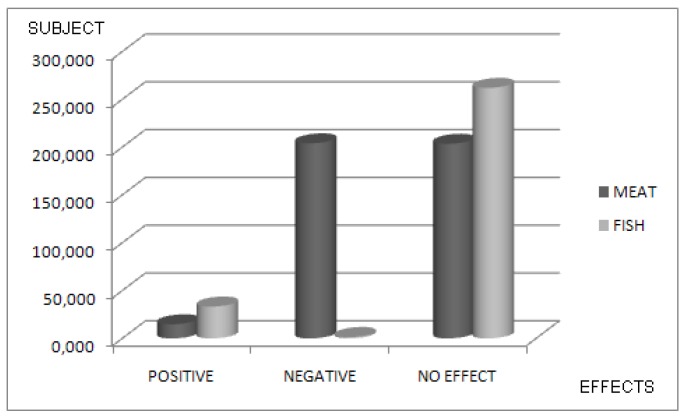
Association between meat and fish dietary patterns and effects on bone mineral density (BMD) or risk of fractures (in % of total study population).

**Figure 3:**
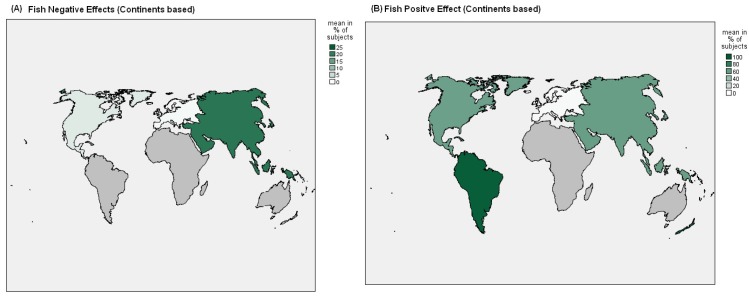
Continents positive effects (on total population) of “meat **(A)** and fish **(B)**” dietary patterns on BMD or risk of fractures.

**Figure 4:**
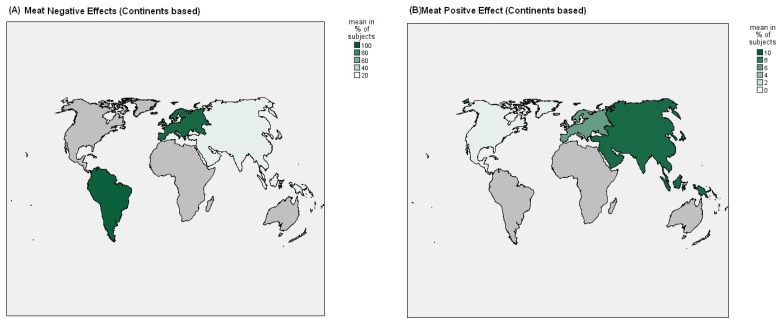
Continents negative effects (on total population) of “meat **(A)** and fish **(B)**” dietary patterns on BMD or risk of fractures.

Figure 2, Figure 3 and Figure 4 showed Nation and Continent wide positive and negative effects of FishDiet and MeatDiet.

In 10 studies [7,25,35,36,38,43,44,47,49,55] conducted in European countries (four in UK, two in Portugal, one in Denmark, three in Netherlands, two in Greece, and one in Germany, Italy, Norway and Spain), in relation to dietary patterns that include meat consumption, positive effects were observed in 12,897 subjects (6.2%) (Netherlands and UK), negative effects were seen in 191,259 subjects (92.7%) (Netherlands, Greece, Germany, Italy, Norway, UK and Spain), and no significant effects were observed in 2226 subjects (1.1%) (Portugal, Denmark and Greece).

In relation to dietary patterns that include FishDiet products, positive effects were observed in 8576 subjects (3.3%) (Netherlands, UK and Greece), adverse effects were observed in 2464 subjects (1%) (UK), and no significant effects were observed in 245,236 subjects (95.7%) (Portugal, Denmark, Netherlands, Greece, Germany, Italy, Norway, UK and Spain). In 13 studies [22,27,28,29,30,31,37,45,47,51,52,53,54] conducted in Asia in relation to meat consumption as a dietary pattern, positive effects were seen in 1458 subjects (9.6%) (China and Japan), adverse effects were seen in 4129 subjects (27%) (South Korea), and no significant effects were seen in 9659 subjects (63.4%) (South Korea, China and Japan). In relation to FishDiet, positive effects were seen in 14,969 subjects (59.9%) (South Korea, China and Japan), adverse effects in 5199 subjects (20.8%) (South Korea), while no significant effects were observed in 4826 subjects (19.3%) (Iran, China and Japan). In nine studies [21,23,24,31,33,41,48,50,56] conducted in North America (seven in USA and two in Canada), positive effects were observed in 1357 subjects (1%) (USA) in relation to MeatDiet and no significant effects were observed in 143,480 subjects (99%) (USA and Canada). No adverse effects in relation to MeatDiet were observed in these studies. In relation to FishDiet, positive effects were seen in 16,476 subjects (57.5%) (USA and Canada), adverse effects were seen in 907 subjects (3.2%) (USA), and no significant effects were observed in 11,272 subjects (39.3%) (USA). In three studies [32,40,42] conducted in South America (two in Brazil, and one in Mexico), no positive effects were observed in relation to MeatDiet, adverse effects were observed in 6915 subjects (96.4%) (Mexico), and no significant effects were observed in 258 subjects (3.6%) (Brazil).

In relation to FishDiet, positive effects were observed in 6915 subjects (97.8%) (Mexico) and no significant effects were observed in 156 subjects (2.2%) (Brazil). No adverse effects in relation to FishDiet were observed in these studies. In three studies [34,39,46] conducted in Australia, on MeatDiet, no positive effects were observed in relation to MeatDiet, adverse effects were observed in 1709 subjects (62.5%), while no significant effects were seen in 1024 subjects (37.5%). In relation to FishDiet, positive effects were seen in 2733 subjects (100%), with no adverse effects observed.

## 4. Discussion

The objective of this systematic review was to evaluate the impact of consumption of animal proteins (derived from fish and meat) on bone metabolism. We aimed to investigate the effects of FishDiet and MeatDiet on BMD or risk of fractures. The review included 37 clinical trials and 432,924 subjects.

### 4.1. The Relevant Data

This review suggests that FishDiet and Meat Diet as a dietary pattern were not associated with an increase/decrease in BMD or Risk of Fracture in 48.2% of subjects with MeatDiet and in 86.5% of subjects with FishDiet. This data was obtained adding all the subjects included in the studies with no statistical significance between the pattern “FishDiet or MeatDiet” and BMD or RF (in terms of OR or RR). These results are in accordance with a recent updated review of the literature which shows that a higher intake of animal protein is not harmful to bone, even though it was once thought that the acid generating components of a high protein diet were detrimental to bone [57].

### 4.2. Negative Effects and Non-Compliance to Mediterranean or Asian Diet

Negative effects on bone were observed in 2.7% of subjects of FishDiet and in 47.9% of subjects of MeatDiet. Major negative effects of MeatDiet were found in a higher number of subjects located in the Netherlands, Greece, Germany, Italy, Norway, the UK and Spain who do not sustain a Mediterranean diet (92.7%); in Korea (27.1%); in Brazil and Mexico (96.4%); and in Australia (62.5%). Firstly, this may be explained by the higher saturated fat content found in red meat compared to other animal protein sources. Saturated fat has been shown to have detrimental effects on bone health in adults, possibly by reducing calcium absorption from the intestine, reducing bone formation, and enhancing bone resorption.

However, several studies suggest that the positive effect of protein intake on bone health may be enhanced by greater calcium intake, perhaps because of increased absorption of calcium [58,59,60,61]. Secondly, as indicated in Figure 3, subjects with MeatDiet were associated with a non-compliance to Mediterranean or Asian diet. As suggested by Maurer et al., a Western-type diet is associated with osteoporosis and calcium nephrolithiasis [62]. Based on observations that calcium retention and inhibition of bone resorption result from alkali administration, it is assumed that the acid load inherent in this diet is responsible for increased bone resorption and calcium loss from bone [63].

As regards the situation in Europe, as suggested by the “Framingam study”, the individuals in the processed protein foods cluster (high percentage of protein intake from cheese, processed meat, sweet baked products, pizza and French fries, snacks and white grains) presented with lower BMD compared to other clusters [64]. Processed meat is also high in sodium. High sodium diets have been shown to alter calcium metabolism and to increase bone resorption in postmenopausal women [65,66]. According to our results on the effects of meat consumption, conspicuous differences are observed in Europe regarding the incidence of osteoporosis, the lowest incidence being reported in the Mediterranean area. In fact, lower negative effects were reported in the Greek population due to their highest adherence to a Mediterranean diet [6,67].

### 4.3. The Positive Effects of FishDiet: Why and What?

The 11% of the subjects associated to FishDiet showed an increase of BMD and a decrease of risk of fractures. The highest positive effects were found in Asia (59.9%) (South Korea, China and Japan), North America (57.5%) (USA and Canada) and South America (97.8%) (Mexico). The fewest negative effects were reported in Australia. Fish and sea-fish potentially have a positive role in BMD mainly due to the well-known anti-inflammatory effects of *n*-3 fatty acids (FAs). Both pro-inflammatory and anti-inflammatory cytokines and hormones interact to regulate osteoblast and osteoclast differentiation and activity [68]. A beneficial interaction between calcium and *n*-3 FAs is plausible based on work done mainly in animal and in vitro models suggesting up-regulation of duodenal calcium absorption and decreased calcium excretion with treatment of *n*-3 FAs [69]. An interesting study by Kontogianni et al. (2009) described in a sample of adult Greek women that adherence to a dietary pattern close to the Mediterranean diet was positively related to BMD, suggesting the potential bone-preserving properties of this pattern through adult life [47].

As regards to the Asian Diet, the Asian population, whose soy and fish intake is higher compared to Western populations, shows a significantly lower incidence of osteoporotic fracture. In fact, several meta-analysis have revealed that supplementation of soy isoflavones with omega 3 improve bone health status in women [70].

### 4.4. Limitation of This Study

This study includes several limitations. The lack of studies performed in other parts of the world such as Africa, Russia, and large parts of Europe may also represent a limitation of our study. The interpretation of summary statistics of data presents some limitations related to methodological issues of the studies included. These studies come from different research environments and use different methods of assessment (such as the variation, over time, of BMD, Risk Ratio, etc.).

Finally, another limitation is that the assessment of bone status in terms of BMD variation or Risk of fractures, often used different adjustment methods, and some did not provide the adjusted rates.

## 5. Conclusions

This study suggests that protein intake from fish or meat is not harmful to bone. In particular, negative effects on bone linked to fish dietary pattern are almost null. As regards to meat dietary patterns, negative effects on bone were associated with meat consumption in the context of a Western diet but not in Mediterranean and Asian Diets.

## Figures and Tables

**Table 1:** Study characteristics.

References	Number of Participants	Age (Years)	Country	Duration of the Study	Dietary Patterns Association with Osteoporosis Outcome Results		Conclusions	Summary of Effect on BMD	Study Design (Level of Evidence)
					Meat	Fish			
Langsetmo, L. et al. (2011) [21]	5188 subjects (1649 men; 3539 women)	≥50	Canada	2 years	Energy-dense factor (EDF) (representing energy-dense foods such as processed meat) are associated with HRs for risk of fractures of: 1.01 (*p* = NS) in women and of 1.08 (*p* = NS) in men.	NR	The intake of processed meat was not associated with fractures.	Meat: no effect on BMD; Fish: NR.	Retrospective cohort study
Karamati, M. et al. (2014) [22]	151 women	60.3 (59.1–61.6)	Iran	3 years	NR	Pattern 2: includes fish intake. Pairwise difference between Lumbar spine: −0.01 g/cm^2^ (*p* = NS); Femoral neck: 0.01 g/cm^2^ (*p* = NS).	Pattern 2: (fish intake) was not associated with lumbar spine or femoral neck BMD.	Meat: NR; Fish: no effect on BMD.	Cross-sectional study
Langsetmo, L. et al. (2010) [23]	6539 subjects (1928 men; 4611 women)	Men: 58.8 (±13.5) women: 61.2 (±12.2)	Canada	5 years (secondary outcome); 2 years (primary outcome)	Energy dense food (included meat). R^2^ for dietary patterns and energy intake as predictors of femoral neck BMD (g/cm^2^). The parameter estimates are for each 1 SD increase of the nutrient dense factor score, the energy dense factor score, the difference between energy dense and nutrient dense factor score, and the log-transformed energy intake (1 SD is roughly 36% change in energy intake). *p*-Values for null hypothesis (from top to bottom). Adult Men: *p* = NS. Older Men: *p* = 0.007 (decrease 1 SD with meat). Premenopausal Women: *p* = NS. Postmenopausal Women: *p* = 0.032 (decrease 1 SD with meat).	Nutrient dense score food (included fish). R^2^ for dietary patterns and energy intake as predictors of femoral neck BMD (g/cm^2^). The parameter estimates are for each 1 SD increase of the nutrient dense factor score, the energy dense factor score, the difference between energy dense and nutrient dense factor score, and the log-transformed energy intake (1 SD is roughly 36% change in energy intake). *p*-values for null hypothesis (from top to bottom). Younger Men: *p* = 0.028 (increase 1 SD with fish); Older men: *p* = NS. Premenopausal Women: *p* = NS. Postmenopausal Women: *p* = NS.	Fish increased BMD in younger men. In older men Meat decreased BMD in older men and in postmenopausal women.	Meat: decreased BMD; Fish: increased BMD.	Longitudinal cohort study
Wosje K. et al. (2010) [24]	325 children	3.8–7.8	USA	4 years	Pattern 1: (meat, poultry, processed meat) high intake of meat. Bone mass increase during the quartile in 1,3 and 4 year (*p* < 0.01).	Pattern 2: high intake of fish. Bone mass increase during the quartile in year 1 and 3 (*p* < 0.01) and decrease during the quartile 4.	Pattern 1 (meat) was significantly associated with higher bone mass. Pattern 2 (fish) data related to bone mass were contradictory	Meat: increased BMD; Fish: no effect on BMD.	Longitudinal study
Fairweather-Tait S.J. et al. (2011) [25]	2464 women	56.3 (±11.9)	UK	11 years	Traditional English pattern score (high intake of fish): Spine: BMD ∆: −0.035 g/cm^2^ (*p* < 0.05); Total hip: BMD ∆: −0.039 g/cm^2^ (*p* < 0.01); Hip Neck: BMD ∆: −0.055 g/cm^2^ (*p* < 0.01).		High intakes of fried fish, fried potatoes, legumes (e.g., baked beans), red and processed meat, vegetables was associated with a lower BMD.	Meat: decreased BMD; Fish: decreased BMD.	Co-twin control study
Shin S. et al. (2013) [26]	3735 women	64.1 (±9.5)	South Korea	3 years	Factor 1: meat consumption. CC among Factor 1 and BMD: Total femur: 0.005 CC; Trochanter: 0.008 CC; Intertrochanter: 0.004 CC; Femoral neck: 0.003 CC; Ward: 0.005 CC; Lumbar spine: 0.031 CC; (*p* = NS) Risk for osteoporosis of the femoral neck and lumbar spine across the quintile (Q) categories in factor 1: Femoral neck odds ratio (OR): 1.01 (*p* = NS); Lumbar spine: OR: 0.72 (*p* = NS).	Factor 3: Seaweed consumption. Correlation coefficients (CC) among Factor 3 and bone mineral density (BMD): Total femur 0.006 CC; Trochanter 0.011 CC; Intertrochanter −0.009 CC; Femoral neck −0.014 CC; Ward −0.040 CC; Lumbar spine −0.040 CC; Risk for osteoporosis of the femoral neck and lumbar spine across the quintile (Q) categories in factor 3: Femoral neck odds ratio (OR): 0.70 (*p* = NS); Lumbar spine: OR: 0.94 (*p* = NS).	Seaweed pattern (Factor 3) had a 40% higher risk of osteoporosis in the lumbar spine.	Meat: no effect on BMD; Fish: decreased BMD.	The Korea National Health and Nutrition Examination Survey (KNHANES: nationwide cross-sectional survey)
Shin S. et al. (2015) [27]	1818 subjects (716 men; 1102 women)	46.4 (±12.3)	South Korea	2 years and 6 month	Factor 2: meat consumption (Meat/poultry/processed meats). Correlation coefficients (CC) among Factor 2 and BMD: Whole arm (g/cm^2^) −0.048 * CC; Whole leg (g/cm^2^): −0.041 CC; Whole pelvis (g/cm^2^): −0.020 CC; Whole spine (g/cm^2^): −0.023 CC; Whole body (g/cm^2^): 0.035 * CC.	Factor 1: fish Consumption. CC among Factor 1 and BMD: Whole arm (g/cm^2^) 0.088 *** CC; Whole leg (g/cm^2^) 0.050 * CC; Whole pelvis (g/cm^2^): 0.038 CC; Whole spine (g/cm^2^): 0.045 CC; Whole body (g/cm^2^): 0.017 CC.	The dietary pattern characterized by the consumption of fish and shellfish was significantly associated with whole-arm BMD only and not with other BMD measurements.	Meat: decreased BMD; Fish: increased BMD.	Healthy Twins Cohort, cross-sectional survey
Park S.J. e al. (2012) [28]	1464 women	58.8 (±6.7)	South Korea	4 years	Factor 3 (Western diet): meat consumption: Radius RR (Risk Ratio): 1.46 (*p* < 0.05) Tibia RR: 1.46 (*p* = NS)	Factor 1 (Traditional diet): fish and seaweed consumption: Radius RR (Risk Ratio): 1.46 (*p* < 0.05); Tibia RR: 1.82 (*p* < 0.05).	Traditional diet with high intake of fish and Western with high intake of meat dietary patterns were associated with greater risk for osteoporosis in postmenopausal Korean women.	Meat: decreases BMD; Fish: decreases BMD.	The Korean Genome and Epidemiology Study (KoGES) is a longitudinal cohort study
Go G. et al. (2014) [29]	847 women	NR	South Korea	1 year	Food group with intake of meat (excluding dairy products, and including grain, vegetables and fruits): GMdVF (Grain, Meat, Dairy, Vegetable, Fruit Capital letter indicates eating a certain amount from the food group; lower case letter means not eating a certain amount from the food group): Normal (*n* = 136): GMdVF: 73 (53.7% n. of subject); Osteopenia (*n* = 413): GMdVF: 216 (53.0%); Osteoporosis (*n* = 298): GMdVF: 166 (59.5%).	NR	Meat consumption does not increase BMD. High number of subjects in group of osteoporosis versus normal (59% vs. 53.7%)	Meat: decreases BMD; Fish: NR.	The Korea National Health and Nutrition Examination Survey (KNHANES: nationwide cross-sectional survey)
Chan R. et al. (2015) [30]	2724 women	71.8 (±4.8)	Hong Kong	2 years	Factor 3: (Meat-Fish) OR: 0.86 (IC 95%: 0.59–1.24) (*p* = NS)	Factor 3: (Fish-meat) OR: 0.86 (IC 95%: 0.59–1.24) (*p* = NS)	There was no association of “meat-fish” pattern with incident frailty	Meat: no effect on BMD; Fish: no effect on BMD.	Prospective cohort study
Choi E. et al. (2016) [31]	9812 women	60	South Korea United States	3 years	NR	In NHANES: *p* = NS; In KNHANES: *p* < 0.05; Correlation coefficients (CC) between bone mineral density (g/cm^2^) of: Total femur (g/cm^2^) men: 0.0748 CC; women: 0.1611 CC; Femoral neck (g/cm^2^) men: 0.0768 CC; women: 0.1806 CC; Lumbar spine (g/cm^2^) men: 0.0465 CC; women: 0.1630 CC.	A positive association between the consumption of fish and shellfish and bone health among men and postmenopausal women over 50 years old in Koreans but not in Americans	Meat: NR; Fish: increases BMD.	KNHANES and the NHANES
De Franca N.A.G. et al. (2016) [32]	156 women	68.4 (±9)	Brazil	3 years	Meat included in “Red meat and refined cereals” dietary pattern Factor-loading matrix in red meat pattern for meat; 0.666 score Results of adjusted linear regression analysis (β-coefficient), and 95% confidence interval (95% CI) of the dietary patterns (score values) and body mineral density (g/cm^2^)): Lumbar spine (β:−0.094) (95% CI: −0.031 to 0.010) Femoral neck (β: −0.005) (95% CI IC: −0.016 to 0.015) Total femur (β:0.038) (95% CI: −0.014 to 0.022) Total body ( β:−0.019) (95% CI: −0.023 to 0.018) (*p* = NS)	Fish included in “Red meat and refined cereals” dietary pattern. Factor-loading matrix in red meat pattern for fish: −0.472 score. Results of adjusted linear regression analysis (β-coefficient), and 95% confidence interval of the dietary patterns (score values) and body mineral density (g/cm^2^): Lumbar spine (β:−0.094) (95% CI: −0.031 to 0.010); Femoral neck (β:−0.005) (95% CI: −0.016 to 0.015); Total femur (β: 0.038) (95% CI: −0.014 to 0.022); Total body (β:−0.019) (95% CI: −0.023 to 0.018); (*p* = NS)	No effects on BMD were observed with meat and fish consumption.	Meat: no effect on BMD; Fish: no effect on BMD.	Cross-sectional study
Nieves J.W. et al. (2010) [33]	125 women	22.1 (±2.6)	USA	2 years	Dietary Pattern 3 (high animal proteins, high fat, low fruit and vegetables, low fiber): Dietary Pattern 3 HR:1.06 (95% CI: 0.54–2.09) (*p* = NS) Dietary Pattern 4 (high protein) are associated with HR of risk of fractures of: Dietary Pattern 4 HR:1.54 (95% CI: 0.31–7.48) (*p* = NS) Animal protein (SD) g/day/kg body Weight and Whole-body BMD (g/cm^2^/year ± SE) 0.00602 ± 0.00219 (*p* < 0.01)		Protein intake, specifically animal protein, was related to small but significantly greater increases in total body bone mass.	Meat: increases BMD Fish: increases BMD	Prospective cohort study
McNaughton S.A. et al. (2011) [34]	527 women	18–65	Australia	10 years	Pattern 1 (Sausages and processed meat), Factor loading: 0.33 score BMC (g/cm^2^) β: −15.07 (*p* < 0.05) Hip BMD (g/cm^2^) β: 0.0013 (*p* = NS) Lumbar spine BMD (g/cm^2^) β:−0.0017 (*p* = NS) Pattern 2 (red meat), Factor loading: 0.27 score BMC (g/cm^2^) β: 3.14 (*p* = NS) Hip BMD (g/cm^2^) β: −0.0009 (*p* = NS) Lumbar spine BMD (g/cm^2^) β: −0.0017 (*p* = NS)	Pattern 3 (Fish) Factor loading: 0.23 score; BMC (g/cm^2^) β: 4.60 (*p* = NS); Hip BMD β: −0.0006 (*p* = NS); Lumbar spine BMD (g/cm^2^) β: −0.0001 (*p* = NS); Pattern 4 (Seafood) Factor loading: 0.48 score; BMC (g/cm^2^) β: 15.20 (*p* = NS); Hip BMD (g/cm^2^) β: 0.0022 (*p* < 0.05); Lumbar spine BMD (g/cm^2^) β: 0.0037 (*p* < 0.05).	Pattern 1(Sausages and processed meat) was inversely associated with total body BMC. Pattern 4 (Seafood) was directly associated with regional BMD and total BMC	Meat: decreases BMD Fish: increases BMD	Cross-sectional study
Monjardino T. et al. (2014) [35]	1023 subjects (474 boys; 549 girls)	13–17	Portugal	2 years	MD (Mediterranean diet) pattern: Meat Girls annual BMD variation (mg/cm^2^ per year): 0.028 (*p* = NS) Boys annual BMD variation (mg/cm^2^ per year): −0.012 (*p* = NS) DASH diet (Dietary Approaches to Stop Hypertension) Girls annual BMD variation (mg/cm^2^ per year): −0.002 (*p* = NS) Boys annual BMD variation (mg/cm^2^ per year): −0.026 (*p* = NS)	MD pattern: Fish; Girls annual BMD variation (mg/cm^2^ per year): 0.028 (*p* = NS); Boys annual BMD variation (mg/cm^2^ per year): −0.012 (*p* = NS); DASH diet (Dietary Approaches to Stop Hypertension): Girls annual BMD variation (mg/cm^2^ per year): −0.002 (*p* = NS); Boys annual BMD variation (mg/cm^2^ per year): −0.026 (*p* = NS).	The selected dietary patterns may not capture the elements of diet that are truly important in determining adolescent bone quality	Meat: no effect on BMD; Fish: no effect on BMD	Epidemiological Health Investigation of Teenagers in Porto (EPITeen population based cohort)
Monjardino T. et al. (2015) [36]	1007 subjects (543 girls; 464 boys)	13–17	Portugal	2 years	Lower intake (red meat): Girls annual BMD variation (mg/cm^2^ per year): −0.381 (*p* = NS); Boys annual BMD variation (mg/cm^2^ per year): 0.333 (*p* = NS)	Lower intake (fish): Girls annual BMD variation (mg/cm^2^ per year): −0.381 (*p* = NS); Boys annual BMD variation (mg/cm^2^ per year): 0.333 (*p* = NS).	There were no consistent associations between dietary patterns and forearm BMD in adolescents.	Meat: no effect on BMD; Fish: no effect on BMD.	Epidemiological Health Investigation of Teenagers in Porto (EPITeen population based cohort)
Zeng F.F. et al. (2013) [37]	581 subjects (148 men; 433 women)	71 (±7)	China	3 years	Healthy Dietary Pattern (Poultry): OR of Hip Fractures (g/cm^2^) for Tertiles: (T3 vs. T1) OR: 0.42 (95% CI: 0.24-0.73), (*p* < 0.01) Prudent Dietary Pattern (Red meat): OR of Hip Fractures (g/cm^2^) for Tertiles: (T3 vs. T1) OR: 0.51 (95% CI: 0.28–0.90),(*p* < 0.05) Traditional Dietary Pattern (Processed meat, animal organ meat): OR of Hip Fractures (g/cm^2^) for Tertiles: (T3 vs. T1) OR: 0.83 (95% CI: 0.49–1.43), (*p* = NS); High Fat Dietary Pattern (Red meat, Poultry, Animal organ meat): OR of Hip Fractures (g/cm^2^) for Tertiles: (T3 vs. T1) OR: 2.25 (95% CI: 1.38–3.69), (*p* < 0.01)	Healthy Dietary Pattern (Freshwater fish): OR (Odds Ratio) of Hip Fractures (g/cm^2^) for Tertiles: (T3 vs. T1) OR: 0.42 (95% CI: 0.24–0.73) (*p* < 0.01); Prudent Dietary Pattern (shellfish, sea fish, processed fish): OR of Hip Fractures (g/cm^2^) for Tertiles: (T3 vs. T1) OR: 0.51 (95% CI: 0.28–0.90) (*p* < 0.05); Traditional Dietary Pattern (shellfish, processed fish): OR of Hip Fractures (g/cm^2^) for Tertiles: (T3 vs. T1) OR: 0.83 (95% CI: 0.49–1.43) (*p* = NS); High Fat Dietary Pattern (shellfish) OR of Hip Fractures (g/cm^2^) for Tertiles: (T3 vs. T1) OR: 2.25 (95% CI: 1.38–3.69) (*p* < 0.01)	The findings suggest that dietary patterns that feature a high intake of fish and low-fat poultry and a low intake of saturated fat may protect against hip fracture.	Meat: increases BMD Fish: increases BMD	1:1 matched case-control study
Petersen S.B. et al. (2015) [38]	53,922 children	<16	Denmark	6 years	Western (meat): HRs:1.03 (*p* = NS) Traditional (meat–poultry): HRs: 1.00 (*p* = NS).	Seafood (fish–shellfish): HRs: 0.94 (*p* = NS).	There were indications that maternal Western diet was associated with offspring forearm fractures. However, it was not possible to identify any single food item in the Western pattern that appeared to be of importance for offspring forearm fracture risk.	Meat: no effect on BMD Fish: no effect on BMD	Prospective study (Danish National Birth Cohort (DNBC))
van den Hooven E.H. et al. (2015) [39]	1024 young adults	14–20	Australia	2 years	Pattern 2 (high-protein, low-calcium, low-potassium): Factor loading: Meat 0.24; Poultry 0.36; Red meat 0.42; Processed meat 0.29 BMD (mg/cm^2^): −0.2 (*p* = NS); BCM (g): −0.5 (*p* = NS).	Pattern 1 (high-protein, high-calcium, high-potassium) Factor loading: Fish 0.18; BMD (mg/cm^2^): 8.6 (*p* < 0.05); BCM (g): 21.9 (*p* < 0.05).	A dietary pattern characterized by high intake of protein and low intakes of calcium and potassium was not associated with later bone outcomes. A dietary pattern characterized by high-protein, high-calcium, high-potassium was associated with higher BMD and BMC	Meat: no effect on BMD Fish: increases BMD	Longitudinal study based on Western Australian Pregnancy Cohort (Raine) Study
Silva T.R. et al. (2015) [40]	99 women	55.2 (±4.9)	Brazil	2 years	OR for low bone mass: Meat and eggs (<96 g/day): OR 2.30 (*p* = NS).	NR	Meat intake did not interfere with BMD, but participants were mostly sedentary	Meat: no effect on BMD Fish: NR	Cross-sectional study
Haring B. et al. (2016) [41]	796 women	63.6 (±7.4)	USA	5 years	Mediterranean Diet (aMED) (red and processed meats): HRs: 0.80 (*p* = NS); Dietary Approaches to Stop Hypertension (DASH) (Red and processed meat): HRs: 0.89 (*p* = NS).	aMED (Fish) Hazard ratios (HRs): 0.80 (*p* = NS) Healthy Eating Index 2010 (HEI-2010) (seafood) HRs: 0.87 (*p* = NS) Alternate Healthy Eating Index 2010 (AHEI-2010) (long-chain ω-3 polyunsaturated fatty acids, polyunsaturated fatty acids) Hazard ratios (HRs): 0.94 (*p* = NS)	There were no consistent associations between dietary patterns and BMD.	Meat: no effect on BMD; Fish: no effect on BMD.	Women’s Health Initiative observational study (WHI-OS)
Denova-Gutiérrez E. (2016) [42]	6915 subjects (1948 men; 4967 women)	20–80	Mexico	NR	Westernized dietary pattern (red meat): Odds ratios (OR): Total BMD (g/cm^2^): Q2 1.54; Q5 1.74 (*p* < 0.05); Hip BMD (g/cm^2^): Q2 1.40; Q5 1.91 (*p* < 0.01); Spine BMD (g/cm^2^): Q2 1.47; Q5 1.61(*p* < 0.05).	“Dairy and fish” dietary pattern (Fish and sea food) Odds ratios (OR) Total BMD (g/cm^2^): Q2 0.69; Q5 0.51 (*p* < 0.001) Hip BMD (g/cm^2^): Q2 0.99; Q5 0.86 (*p* = NS) Spine BMD (g/cm^2^): Q2 0.87; Q5 0.69 (*p* < 0.001)	A “dairy and fish” dietary pattern may contribute to better BMD. In contrast, a Westernized dietary pattern was significantly associated with higher likelihood of low BMD.	Meat: decreases BMD Fish: increases BMD	Cross-sectional analysis (Health Workers Cohort Study (HWCS))
De Jonge E.A.L. et al. (2016) [43]	5144 men and women	≥55	Netherlands	11 years	Traditional dietary pattern (meat): BMD of the femoral neck: 0.01 g/cm^2^ (*p* = NS); Processed dietary pattern (processed meat): BMD of the femoral neck: −0.03 g/cm^2^ (*p* = NS); Health dietary pattern (poultry): BMD of the femoral neck: 0.04 g/cm^2^ (*p* = 0.01).	Health dietary pattern (fish): BMD of the femoral neck: 0.04 g/cm^2^ (*p* = 0.01).	Health dietary pattern has benefits for BMD; in contrast, adherence to a Processed dietary pattern may pose a risk for low BMD.	Meat: increases BMD Fish: increases BMD	The Rotterdam Study (population-based cohort study)
Hardcastle A.C. et al. (2011) [44]	3236 women	55.1 (±2.2)	Scotland	9 years	Healthy pattern (meat). Multiple linear regression associated with the two bone resorption markers, fPYD/Cr (free deoxypyridinoline expressed relative to creatinine) and fDPD/Cr (free pyridinoline expressed relative to creatinine), HRT use and menopausal status. fPYD/Cr: Unstandardised β: 3.42 (95% CI: 3.13, 3.72) (*p* < 0001); fDPD/Cr: Unstandardised β: 2.07 (95% CI: 1.76, 2.39) (*p* < 0001).	Healthy pattern (fish). Multiple linear regression associated with the two bone resorption markers, fPYD/Cr (free deoxypyridinoline expressed relative to creatinine) and fDPD/Cr (free pyridinoline expressed relative to creatinine), HRT (hormone replacement therapy) use and menopausal status. fPYD/Cr: Unstandardised β: 3.42 (95% CI: 3.13, 3.72) (*p* < 0001); fDPD/Cr: Unstandardised β: 2.07 (95% CI: 1.76, 2.39) (*p* < 0001).	White meat, white and oily fish and dairy products contain nutrients that are associated with good bone health.	Meat: increases BMD Fish: increases BMD	Cross-sectional study
Mu M. et al. (2014) [45]	1319 men	18.1 (±1.2)	China	1 month	Animal Protein Pattern: Meat (Lard, fat and lean meat) (Chicken, duck, goose): Hazard ratios (HR): 1.04 (*p* = NS)	Animal Protein Pattern: Fish (Carp, grass carp, silver carp, herring, shrimp) (Kelp laver, sea fish, seaweed) Hazard ratios (HR): 1.04 (*p* = NS)	The animal protein pattern was not associated with a decreased or increase risk of osteopenia or osteoporosis	Meat: no effect on BMD Fish: no effect on BMD	Cross-sectional study
Melaku Y.A. et al. (2016) [46]	1182 men and women	median 62 years	South Australia	11 years	Pattern 2 (“Western pattern”) includes high levels of processed and red meat, poultry: PR for the association between tertiles of food patterns and low bone mineral density. PR: 1.68 (95% CI: 1.02–2.77) (*p* < 0.05)	Pattern 1 (“prudent pattern”) includes fish PR for the association between tertiles of food patterns and low bone mineral density. PR: 0.52 (95% CI: 0.33–0.83) (*p* < 0.01).	Western pattern characterized by high intakes of processed and red meat was inversely associated with BMD Prudent pattern characterized by high intakes of fish was associated with higher BMD	Meat: decreases BMD Fish: increases BMD	The North West Adelaide Health Study (NWAHS)
Kontogianni M.D. et al. (2009) [47]	196 women	48 (±12)	Greek Greece	NR	A pattern characterized by high consumption of poultry (coefficient score 0.855) (component 4) BMD (lumbar bone mineral density) (g/cm^2^) β: 0.054 (*p* = NS)	A pattern characterized by high consumption of fish (coefficient score 0.867) (component 3); lumbar BMD (g/cm^2^) β: 0.185 (*p* < 0.05)	A dietary pattern characterized by high consumption of fish and low red meat intake was associated with higher BMD	Meat: no effect on BMD Fish: increases BMD	Cross-sectional study
Tucker K.L. et al. (2002) [48]	907 women and men	Men 75.1 (±4.9) Women 75.3 (±4.8)	USA	2 years	“Meat, dairy, and bread” group (*n* = 313) including *p* < 0.05: red meat, chicken BMD (±SE): Femoral neck 0.86 g/cm^2^ (*p* = 0.001); in men and in women 0.74 g/cm^2^ (*p* = NS); “Meat and sweet baked products” group (*n* = 260) including% *p* < 0.05: red meat, processed meat Adjusted mean (±SE) bone mineral density (BMD) “Sweet baked products” group (*n* = 69) including% *p* < 0.05: chicken Adjusted mean (±SE) bone mineral density (BMD)	“Meat, dairy, and bread” group (*n* = 313) including% *p* < 0.05: fish Adjusted mean (±SE) BMD “Sweet baked products” group (*n* = 69) including% *p* < 0.05: fish Adjusted mean (±SE) bone mineral density (BMD) “Candy” group (*n* = 75) including% *p* < 0.05: fish Adjusted mean (±SE) bone mineral density (BMD)	Men with a diet high in fruit, vegetables, and cereal (red meat and processed meat) had significantly greater BMD than did men with other dietary patterns. In contrast, those consuming the most candy (fish) had significantly lower BMD than did most other groups.	Meat: increases BMD Fish: decreases BMD	Longitudinal cohort study (The Framingham Heart Study)
Whittle C.R. et al. (2012) [49]	489 women and men	Men 22.4 (±1.6) Women 22.8 (±1.7)	Northern Ireland	2 years	Factor loading for men Factor 1: “Healthy” included meat dishes: −0.365 CC. BMD for the quintiles (Q) group of Healthy pattern determined by a posteriori principal component analysis. LS BMD (g/cm^2^) Q1–Q5 (*p* = NS). FN BMD (g/cm^2^) Q1–Q5 (*p* = NS). LS BMC (g) Q1–Q5 (*p* = NS). FN BMC (g) Q1–Q5 (*p* = NS); Factor 2: “Traditional” included red meat: 0,398 CC. Included poultry : –0.272 CC. LS BMD (g/cm^2^) Q1–Q5 (*p* = NS). FN BMD (g/cm^2^) Q1-Q5 (*p* = NS). LS BMC (g) Q1–Q5 (*p* = NS). FN BMC (g) Q1–Q5 (*p* = NS); Factor 3: “Refined” included meat dishes: 0,257 CC. LS BMD (g/cm^2^) Q1–Q5 (*p* = NS). FN (Femoral Neck) BMD (g/cm^2^) Q1–Q5 (*p* = NS). LS BMC (g) Q1–Q5 (*p* = NS). FN BMC (g) Q1–Q5 (*p* = NS) Adjusted (*p* < 0.05). Factor loading for women Factor 1: “Healthy” included meat dishes: −0.319 CC. LS BMD (g/cm^2^) Q1–Q5 (*p* = NS). FN BMD (g/cm^2^) Q1–Q5 (*p* = NS). LS BMC (g) Q1–Q5 (*p* = NS). FN BMC (g) Q1–Q5 (*p* = NS). Factor 2: “Traditional” included red meat: 0.299 included poultry: 0.337 LS BMD (g/cm^2^) Q1–Q5 (*p* = NS). FN BMD (g/cm^2^) Q1–Q5 (*p* = NS). LS BMC (g) Q1–Q5 (*p* = NS). FN BMC (g) Q1–Q5 (*p* = NS); Factor 3: “Nuts and Meat” included meat dishes: 0.372 LS BMD (g/cm^2^) Q1–Q5 (*p* = NS); FN (Femoral Neck) BMD (g/cm^2^) Q1–Q5 (*p* = NS) Adjusted (*p* < 0.05). LS BMC (g) Q1–Q5 (*p* = NS). FN BMC (g) Q1–Q5 (*p* = NS) Adjusted (*p* < 0.05).	Factor loading for men:Factor 4 “Social” included white fish: 0.436 CC. LS BMD (g/cm^2^) Q1–Q5 (*p* = NS); FN BMD (g/cm^2^) Q1–Q5 (*p* = NS); LS BMC (g) Q1–Q5 (*p* = NS). FN BMC (g) Q1–Q5 (*p* < 0.05) Adjusted (*p* = NS); Factor loading for women: Factor 1: “Healthy” included white fish: 0,325 CC. LS BMD (g/cm^2^) Q1–Q5 (*p* = NS). FN BMD (g/cm^2^) Q1–Q5 (*p* = NS); LS BMC (g) Q1–Q5 (*p* = NS). FN BMC (g) Q1–Q5 (*p* = NS).	“Refined” group scores (Factor 3 for men) (meat dishes) and “Nuts and Meat” group scores (Factor 3 for women) (meat dishes) were associated with higher FN BMC and in women also FN BMD. “Social” group scores were associated with higher FN BMC but when further adjusted were not significant.	Meat: increases BMD Fish: no effect on BMD	Longitudinal study (The Northern Ireland Young Hearts Project)
Mangano K.M. et al. (2017) [50]	2986 women and men	40.6 (±8.7)	USA	3 years	Red Meat Femoral neck (g/cm^2^) (*n* = 2903) 0.989 ± 0.006 (*p* = NS); Trochanter (g/cm^2^) (*n* = 2903) 0.800 ± 0.006 (*p* = NS); Total femur (g/cm^2^) (*n* = 2903) 1.012 ± 0.006 Lumbar spine (g/cm^2^) (*p* = NS) (*n* = 2831) 1.227 ± 0.009 (*p* = NS); Chicken Femoral neck (g/cm^2^) (*n* = 2903) 1.002 ± 0.006 (*p* = NS); Trochanter (g/cm^2^) (*n* = 2903) 0.806 ± 0.006 (*p* = NS); Total femur (g/cm^2^) (*n* = 2903) 1.022 ± 0.006 (*p* = NS); Lumbar spine (g/cm^2^) (*n* = 2831) 1.233 ± 0.008 (*p* = NS)	Fish: Femoral neck: 1.000 ± 0.006 g/cm^2^ (*n* = 2903) (*p* = NS); Trochanter: 0.805 ± 0.006 g/cm^2^ (*n* = 2903) (*p* = NS); Total femur 1.016 ± 0.007 g/cm^2^ (*n* = 2903) (*p* = NS); Lumbar spine: 1.239 ± 0.009 g/cm^2^ (*n* = 2831) (*p* = NS).	No differences at any BMD site were observed across the protein food clusters in either crude models or adjusted models.	Meat: no effect on BMD; Fish: no effect on BMD	The Framingham Third Generation Study; Longitudinal cohort study
Monma Y. et al. (2010) [51]	877 women and men	80.7 (±5.2)	Japan	4 years	Factor 2: “Meat” pattern included Pork, beef, ham, liver, Chicken. HR (95% CI) of fall-related fracture in each dietary pattern. T2 (moderately confirmed) HR: 0.36 (95% CI: 0.14–0.96); T3 (confirmed) HR: 0.36 (95% CI: 0.12–1.06) (*p* = NS)	Factor 2: “Meat” pattern included Shellfish, Cuttlefish, Octopus, Shrimp. HR (95% CI) of fall-related fracture in each dietary pattern. T2 (moderately confirmed) HR: 0.36 (95% CI: 0.14–0.96) T3 (confirmed) HR: 0.36 (95% CI: 0.12–1.06) (*p* = NS)	The “Meat” pattern had a tendency towards reduced risk of fall-related fracture.	Meat: increases BMD Fish: increases BMD	Prospective study
Okubo H. et al. (2006) [52]	291 women	40–55	Japan	3 years	Factor 3: “Western” (Processed meats and meats); Q1:0.501–0.006 g/cm^2^ and Q5: 0.482–0.007 g/cm^2^; (*p* = NS)	Factor 1: “Healthy” (Fish and shellfish and processed fish) Q1: 0.476–0.006 g/cm^2^; Q5: 0.498–0.006 g/cm^2^; (*p* < 0.05)	Healthy pattern (fish) had a significantly higher BMD. No significant association was observed in the Western pattern (meat) for premenopausal women.	Meat: no effect on BMD Fish: increases BMD	Japanese Multi-centred Environmental Toxicant Study (JMETS)
Yang Y. et al. (2016) [53]	1590 boys and girls	15.1 (±1.3)	China	NR	“Meat” diet Low Bone Quality OR: T2 OR: 0.911 (95% CI 0.620–0.255); T3 OR: 0.920 (95% CI 0.626–1.354); (*p* = NS)	“Chinese and Western” Low Bone Quality OR: T2 OR: 0.621 (95% CI 0.512–0.832); T3 OR: 0.558 (95% CI 0.414–0.901); (*p* < 0.05)	The risk of low bone mineral quality could be reduced by the Chinese and Western structure.	Meat: no effect on BMD; Fish: increases BMD.	Cross-sectional study
Muraki S. et al. (2007) [54]	632 women	71.8 (±7.5)	Japan	NR	NR	Fish consumption BMD (g/cm^2^) 0.791 ± 0.192 T score −1.73 ± 1.59 (*p* = NS)	Consumption or exclusion of fish in the diet has no significant effect on bone health	Meat: NR Fish: no effect on BMD	Cross-sectional study
de Jonge E.A. et al. (2017) [55]	4028 subjects (1705 men; 2323 women)	Men 66 (61–72) Women 66 (61–73)	Netherlands	NR	Pattern: ”Sweets, animal fat, and low meat”: Osteoporotic fractures HR: 1.10 (95% CI: 1.06–1.15) (*p* < 0.05). HRs represent the difference in instantaneous risk of fracture per 1 z score difference in dietary pattern adherence. Hip fractures HR: 1.10 (95% CI: 1.01–1.19) (*p* < 0.05).	NR	Each z score of adherence to the sweets, animal fat, and low meat pattern was associated with higher bone width	Meat: increases BMD Fish: NR	Cross-sectional associations (Rotterdam Study)
Fung T.T. et al. (2015) [56]	112,845 subjects (38,305 men; 74,540 women)	Women: 30–55; Men: 40–75	USA	2 years	Relative risk (RR) (95% CI) for hip fractures according quintiles of dietary patterns: Prudent pattern (poultry and red meat): Women Q1:1–Q5: RR: 1.14 (95% CI: 0.96–1.36) (*p* = NS); Men Q1:1–Q5: RR: 0.86 (95% CI: 0.64-1.16) (*p* = NS). Western pattern (poultry and red meat): Women Q1:1–Q5: RR: 1.05 (95% CI: 0.87–1.26) (*p* = NS); Men Q1:1–Q5: RR: 1.03 (95% CI: 0.73–1.46) (*p* = NS).	NR	Neither the Prudent nor the Western dietary pattern was associated with risk of hip fractures in postmenopausal women or men over 50 years of age.	Meat: no effect on BMD Fish: NR	The Nurses’ Health Study and the Health Professionals Follow-up Study
Benetou V. et al. (2013) [7]	188,795 subjects (48,814 men; 139,981 women)	48.6 (±10.8)	Germany, Greece, Italy, Netherlands, Norway, Spain, Sweden, UK	8 years	HR for incident hip fracture per indicated increments of intake with 95% CI in overall sample: HR per 1-unit increment: 1.18 (95% CI: 1.06–1.3); men HR per 1-unit increment 1.10 (95% CI: 0.92–1.32); women HR per 1-unit increment 1.14 (95% CI: 0.99–1.31).	HR for incident hip fracture per indicated increments of intake with 95% CI in: overall sample HR per 1-unit increment 0.96 (95% CI: 0.86–1.07) men HR per 1-unit increment 0.89 (95% CI: 0.73–1.09) women HR per 1-unit increment 0.97 (95% CI: 0.85–1.12)	High meat intake was associated with increased hip fracture incidence Higher fish consumption was weakly, although not significantly, associated with lower hip fracture incidence	Meat: decrease BMD; Fish: no effect on BMD	Prospective study

* (*p* < 0.05); *** (*p* < 0.001). CI, confidence intervals. HR, Hazard ratios. CC, Correlation coefficients. FN, Femoral Neck. LS, Lumbar Spine. BMD, bone mineral density. BMC, Bone Mineral Content. ±SE, Adjusted mean. PR, Prevalence Ratio. NHANES, the National Health and Nutrition Examination Survey. OR, odds ratio. MD, Mediterranean diet. HRT, hormone replacement therapy. R^2^, Regression coefficients. NR, not recorded. SD, standard deviation.
